# Living in the Era of an Ideological Climate of Globalisation: A Study of Psychological Sense of Community Among Young and Older Adults in Two Cultures (India and Norway). Challenges for Community Psychology and the Applied Social Sciences

**DOI:** 10.3389/fpsyg.2021.718190

**Published:** 2021-07-28

**Authors:** Nina Kavita H. Bahl, Hilde E. Nafstad, Rolv Mikkel Blakar, Eva Langvik

**Affiliations:** ^1^Department of Research and Development, Clinic of Substance Use and Addiction Medicine, St. Olavs University Hospital, Trondheim, Norway; ^2^Department of Psychology, Norwegian University of Science and Technology, Trondheim, Norway; ^3^Department of Psychology, University of Oslo, Oslo, Norway

**Keywords:** age group, globalisation, meaning systems, neo-liberalism, psychological sense of community, family

## Abstract

How do people describe the psychological sense of community (PSOC) in the present day ideological climate of globalising neo-liberalism, assuming that people are essentially individualistic, that solidarity, social commitment, and citizenship are not natural dispositions, as we all are the lonely citizen? This issue is addressed by a mixed-methods study using semi-structured interviews with two age groups—young and older people—from two different cultures—India (Mumbai) and Norway (Oslo). This two by two design gives the opportunity to analyse people’s meaning systems of PSOC, asking; is there a core meaning system of PSOC shared by people within as well as across cultures? Belongingness and citizenship are continuously formed and negotiated, just at the intersection of two dimensions: culture and historical time. The young and older adult informants often live in different “historical times.” The meaning systems of PSOC were explored and compared by language analyses of words used by the informants. Text search queries were made for 69 words. “Help,”, “care,” “different,” “problem,” and “family” were identified as central for further in-depth qualitative analyses. The word, “family” demonstrated high frequencies of use across sub-samples. There was nothing more relevant for the groups than the family when thinking of PSOC, revealing almost a “prior to society perspective.” PSOC is about being part of families. Simultaneously, we are members of other communities: schools, workplaces, neighborhoods, cities and nations. The informants mentioned such communities, but not often. Feeling part of the family, helping and caring not only the family but also your neighbourhood, local community, or national and global communities are particularly necessary today, as we live in a time where communities, societies, and nations across the world are heavily impacted by the COVID-19 pandemic. In this crisis, it is vital that nobody forgets that we are national and transnational citizens and part of many interrelated social systems. This study points out how community psychology and the applied social sciences can work to strengthen the feelings of connections to other communities, societies, and nations outlining and co-creating transformative multi-level interventions of public policy programmes of inclusion and “we-ness.”

## Introduction

In different ways, our development, health, and wellbeing deeply depend on how we, as citizens, manage to create communities where individual wellbeing is interwoven with the wellbeing of others and collective wellbeing. The concept of psychological sense of community (PSOC) is one of the core concepts within the community psychology to approach this fundamental aspect of life— that we are all parts of communities and are dependent on each other (Sarason, 1986; Brodsky et al., 2002; Prilleltensky, 2010; Mannarini and Rochira, 2014). This project studies and compares the conceptualisations or meaning systems of PSOC of young adult and older adult people within as well as across different cultures.

### Psychological Sense of Community

The phenomenon of PSOC refers to the meaning systems of being part of, of being connected and supported, and the values of caring, of compassionate, and including relationships and communities as well as about social responsibility (Sarason, 1974; McMillan, 1996; Brodsky, 2009; Nowell and Boyd, 2010; Kloos et al., 2012). The conceptualisation of PSOC by McMillan and Chavis (1986) is most widely applied today. Their point of departure is that conceptually, PSOC consists of the following four different dimensions (Chavis et al., 2008): (a) a feeling of belonging and identification with the community (membership); (b) a sense or feeling of having some influence on the community and experiencing an acceptable influence from the community (mutual influence); (c) integration and fulfillment of members needs through the resources of the community and members contribution to the communities needs and resources (fulfillment of needs), and finally, (d) a sense that members of the community share and will continue to share a common history (shared emotional connection). In addition to these core dimensions, the concept includes additional dimensions of affect (positive and negative PSOC) and community references [geographical, relational, and ideal communities (Glynn, 1981; Brodsky et al., 2002; Mannarini and Rochira, 2014)]. Thus, the concept of PSOC is multifaceted and multidimensional. A number of meanings are attached to the concept and it is important to know more about how people at a particular societal and historic time, and being in different periods of life as well as in different cultures think about belonging and being part of groups, communities, and societies (Brodsky, 2009; Mannarini and Fedi, 2009; Barbieri et al., 2014; Li et al., 2014; Stewart and Townley, 2020). Taking as point of departure that PSOC is an important dimension in individual, community and societal well being, this project is studying and comparing young adult and older adult people’s conceptualizations or meaning systems of sense of community within as well as across two different cultures, India and Norway.

### Language, Words and Cultures’ Meaning Systems

Meaning systems are embedded in language and are mirrored in the ways words and concepts are used. Words thereby reflect thoughts and feelings about social phenomena and processes (Blakar, 1979; Rommetveit, 1992; Billig, 1997; Pennebaker et al., 2003; Nafstad et al., 2009; Pennebaker, 2011; Holtgraves, 2014; Formanowicz et al., 2016; Carlquist et al., 2017). Systematic empirical analyses of the usage of even single words can therefore serve as descriptive indicators of societal and psychological phenomena and processes, such as PSOC (Blakar, 1979; Rommetveit, 1992; Billig, 1997; Pennebaker et al., 2003; Nafstad et al., 2009; Moghaddam and Harré, 2010). Languages vary greatly across the world; however, all cultures may use words and concepts for bonding and togetherness (Wierzbicka, 1997; Goddard and Wierzbicka, 2014). At the same time, the words and concepts we use at one point of time change due to historical, cultural, and societal changes with profound consequences on our thinking, feeling, and behaviour, which again have consequences for a good life. Thus, the words we use define our understanding of our socio-cultural reality, thereby providing an impact on our thinking, feeling, and planning of how to live and organise the social life.

### Life Span, Cultural and Historical Meaning Systems, and PSOC

Throughout life, people are exposed to differential historical and cultural contexts, having constantly to shape, adjust, and reorient thoughts, feelings, and behaviour to prevailing meaning systems, ideologies, values, norms, opportunities, and deprivations in their society (Elder, 1974, 1980). Hence young and older people within a culture as well as across cultures have often experienced different values and meaning systems of PSOC, and how to be connected to micro, meso, and macro contexts (Nafstad et al., 2013).

Studies have shown that culture, context, and age have an important role in the conceptualisation and meanings of PSOC of people (Dudgeon et al., 2002; Brodsky, 2009; Mak et al., 2009; Kenyon and Carter, 2011; Barbieri et al., 2014; Li et al., 2014). Research includes studies of the relationships between PSOC and cultural meaning systems like collectivism (Love, 2007; Moscardino et al., 2010); the impact of strong individualism in society (Sarason, 1974; Love, 2007; Nafstad et al., 2009); the role of the family system as a source for PSOC, particularly in collectivistic cultures (Cicognani et al., 2008; Brodsky, 2009; Chiessi et al., 2010; Carrillo et al., 2015), as well as for young people (Moscardino et al., 2010). With respect to age and PSOC, it is often life-span related community transitions, which are in focus on studies related to PSOC. Young adult age, for example, typically represents the end of schooling, moving out of the family, often moving to new communities, and starting up own family (Mahan et al., 2002; Fyson, 2008; Chiessi et al., 2010). Thus, central tasks of young adult life are to acquire new values and learning of new social roles: worker, partner/spouse, parent, and the role of being a citizen with rights and obligations (Arnett, 2002; Colby et al., 2003), often in a new community. In old age, on the other hand, people go through transitions, such as retirement from society, the work community, adjusting lifestyle to lower income and withdrawing, and thereby reducing the social roles and networks (Li et al., 2014; Provencher et al., 2014; Singelenberg et al., 2014). Also, people in their old age often experience changes in health and loss of spouse and friends, wishing now for programmes of assistance, care or, help that might not be there in their community (Phillipson, 1993; Bahl et al., 2017). Thus in both of these two life stages, PSOC of people is at risk for decreasing due to ordinary life stress: in the younger years, if the social context becomes insufficient in satisfying evolving active needs in the process of constructing and adapting to a grown-up life and becoming a citizen with duties and rights (Arnett, 2002; Colby et al., 2003; Cicognani et al., 2014). In old age, social change and alteration of social structures, roles, family, social networks, often diminishing physical skills, and sometimes strong increase in frailty, most probably demand increasingly more individual efforts in order to maintain PSOC and find groups to be a part of and stay socially active (Bahl and Hagen, 2017; Bahl et al., 2017). In the worst case, old people, as all of us today in this global situation of a COVID-19 pandemic, need extensive, integrated community, and municipality-based interventions of help and assistance.

### Psychological Sense of Community and the Globalised World Today

Most emic research on the PSOC of people has so far focussed primarily on the local cultural meaning systems. However, more and more global meaning systems continuously affect local cultural meaning systems. Therefore, the experiences of people with regard to PSOC can no longer be understood only as locally culture-bound (Baars et al., 2006; Torres, 2006; Sokolovsky, 2009; Li et al., 2014). Moreover, as age has seldom been the focus of studies of PSOC, we do not know if people in different life-stages, within one cultural context, share the same meaning systems of PSOC. Due to stronger globalisation throughout life span, people today are also continuously exposed to changing historical and cultural experiences having constantly to shape, adjust, and reorient their thoughts, feelings, and behaviour to the prevailing as well as changing meaning systems, values, opportunities, crisis, and deprivations in the society. Consequently, within one society, people in different life stages, young and older people, can belong to different historical generations (Elder, 1974, 1980). People within the society are thus likely to experience rather different values and meaning systems over time about how to secure health and wellbeing for themselves; for others, family and society are at large (Nafstad et al., 2009). This situation characterised by differential meaning systems can create and shape conflicts and distances between people.

We studied the meaning systems of PSOC of young adults and older adults from two urban contexts, Mumbai in India and Oslo in Norway, assuming that there might be different conceptions about PSOC within as well as across the two cultures. At the same time, people in urban India and Norway live more and more in a similar historic period shaped by neo-liberal values (Harvey, 2005; Nafstad et al., 2009; McDonald et al., 2017), defining their conceptions of what matters in social relations, in life, and in society at large.

### Meaning Systems in India and Norway

In every culture, there is a set of ideas about collectivism and individualism and these two dimensions are of the most profoundly researched dimensions in psychology (Chiu and Hong, 2013). While the core individualism is of the belief that the self is a self-contained independent entity and a social pattern of loosely linked individuals, the essence in collectivism is the conviction of the self as continuously interdependent with some in-group (e.g., the family) and social structures and patterns consisting of closely linked individuals (Markus and Kitayama, 1991; Trafimow et al., 1991; Triandis, 1993, 1995; Hofstede, 2001). These dimensions most probably will manifest themselves differently in different societies, influencing conceptualisations and meanings of PSOC in various ways with consequences for how to live and thereby creating the well-functioning of communities and societies. The horizontal-vertical dimension is also an additional and especially important dimension in the understanding of cultures (Triandis, 2001). Typically, horizontal cultures emphasise equality (e.g., welfare systems and egalitarian values), while vertical cultures emphasise hierarchy (e.g., systems, such as the cast system and competitive values).

In Norway, the individualistic meaning systems have historically co-existed within a welfare ideology valuing social equality, social obligation, equitable distribution of wealth, and quality health care for all (Carlquist et al., 2007). This is reflected in the fact that Norwegian culture often is categorised as a horizontal individualistic, with little appreciation for social hierarchy or competition (Triandis and Gelfand, 1998; Triandis, 2001). Today, however, with the influence of an increasingly powerful globalised neo-liberalistic ideology, also within the Norwegian context, values have changed toward more vertical individualism thereby challenging the implications for social life and citizenship solidarity (Carlquist et al., 2007). These increasingly dominant vertical values, conflicting with the horizontal values, which Norwegian older adults have grown up with, have become the central parts of the discourses of the young adults in the Norwegian context (Türken et al., 2016).

India is a country with complex cultural context as it consists of several states with different cultures and ideologies about how to live (Delle Fave et al., 2016). Biswas-Diener et al. (2012:14) also pointed out that “India is a diverse society, with the largest concentration of Hindus in the world, as well as sizeable Muslim and Christian populations.” Triandis (1996) and Verma and Triandis (1999) categorised the Indian culture as a vertical collectivistic, suggesting that overall, Indian individuals prefer hierarchy within groups and identify themselves more strongly in terms of their in-group relationships, striving to realise shared goals within these relationships. However, today, both the collectivistic and individualistic values co-exist in the Indian contexts (Sinha and Tripathi, 1994; Sinha et al., 2001). Urban areas are particularly exposed to globalised ideologies, and young adults tend to have more individualistic orientations than older adult people (Mishra, 1994; Shah, 2009). As such, urban Indian older adults today most likely have to position themselves in terms of two very different meaning systems, the strictly collectivistic one they grew up in, and the emerging globalised and individualistic value and social practice system.

Thus, around the world young adults’ and old adults’ adjustment and reorientations to such alterations in society certainly have consequences for their conceptualizations and value experiences of PSOC and thereby how they plan and hope to arrange their lives with consequences for own and others’ life, health and well-being.

### The Aims of the Study

Using language analyses, the aims of the present study are to provide a more multifaceted understanding of PSOC and culture in India and Norway; how interactive meaning systems of local and global values within these two cultural contexts most probably are also reflected in the meaning systems of PSOC among two age groups which are at different life-stages that are often characterised with rather heavy and specific ordinary life stress.

The aims of this study are as follows:

(1) As words and expressions are mirroring people’s feeling and thinking we will identify and compare the words and expressions urban young adults and older adults from India and Norway use when describing their own meaning of PSOC;
(2) To map out differences and similarities between the two cultures as well as the two age groups within each culture in the usage of these words and expressions, and finally;
(3) To analyse if and how global cultural meaning systems are currently reflected in the different conceptualisations of PSOC among sub-groups.

To answer these questions, we analyse the language use of the four sub-samples in a mixed-methods design.

## Materials and Methods

**FIGURE 1:**
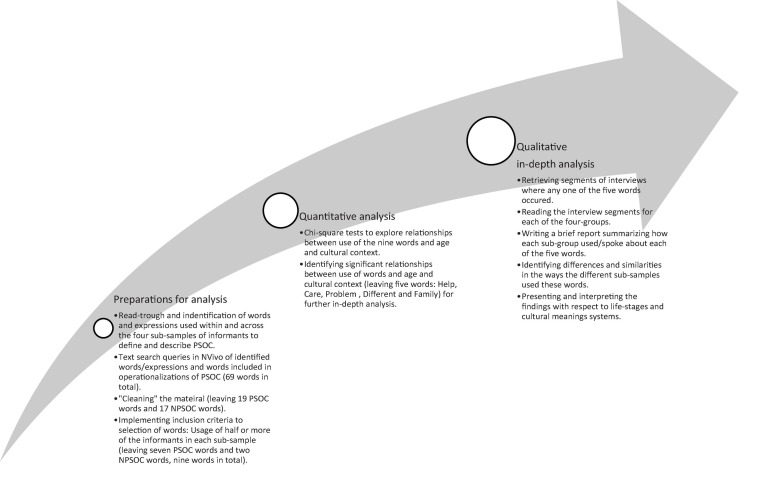
Flowchart of the analysis.

This study used a three-step mixed-methods design to analyse an interview material from four sub-samples (Figure 1). The first step was done to identify words and expressions used within and across sub-samples (Aim 1). As a second step, a quantitative analysis was carried out to explore the relationships between the use of words and age and cultural context (Aim 2). Finally, as the third step, a qualitative analysis was conducted to get an in-depth understanding of how each sub-group spoke about specific words and differences and similarities among sub-samples that used these words (Aims 1 and 2). Both of these aspects were then interpreted with respect to life-stages and cultural meaning systems (Aim 3).

### Oslo and Mumbai as Contexts for Recruitment of Informants

Oslo and Mumbai were purposively chosen as contexts to recruit informants from because these two cities are considered the most globalized contexts in Norway and India, respectively. Oslo is the capital of Norway and holds a population of 697,010 people. With reference to the larger nation, Oslo is seen to be heterogenic with respect to the meaning systems of people: religion, cultural backgrounds, and demographics. A high degree of the internal and cross-border migration of the nation is to Oslo (Statistics Norway, 2021). In addition, there are marked resource and health differences between people in the east and west of Oslo—people in the west have better resources and health (Grøtvedt, 2002). Mumbai is a megacity with over 12 million people and is considered to be the economical capital of India. Due to rapid internal migration, the majority of the “Mumbaikian” population are migrants from other parts of India, and Mumbai is considered a cosmopolitan context, representing the diverse meaning systems in India with respect to religion, culture, and demographics (Bhagat and Jones, 2013). Mumbai is structured into urban (Mumbai city) and (western and eastern) sub-urban areas.

### Informants

Our sample consisted of 44 informants; 10 young adults and 12 older adults from Norway (Oslo) and India (Mumbai), respectively. Participants were recruited by means of a combination of purposeful, convenient, and snowball sampling strategies. To secure a broad sample of older adults in different situations, senior centres in different parts of Oslo, from the east and west, were chosen to recruit older adult informants in Norway (Bahl et al., 2017). In India, we used both the membership lists from Lions Club Mumbai and day care centres in different urban and sub-urban areas of Mumbai to recruit older adult informants in different situations (Bahl and Hagen, 2017). The samples of young adult informants were recruited by convenience sampling (asking individuals randomly passing the first author if they wanted to participate in the study) at university campuses (University of Oslo, Oslo, Norway and Tata Institute of Social Sciences, Mumbai, India) as well as by convenient snowball sampling (asking included informants and acquaintances about additional informants fitting the inclusion criteria of age and residential context). The age of the older adult informants ranged from 60 to 85 years. The age of the young adults ranged from 18 to 28 years (see Table 1 for additional information about the demographical background of the informants). All four sub-samples included an equal number of male and female informants.

**TABLE 1:** Informants’ demographic characteristics.

**Informants**	**Age**	**Relationship status**	**Highest level of education**	**Years of residency**	**Children**	**Informants living with family members**
**Norway**						
Older adults	62–85 years	Six widow(er), three divorced, and three married	Non-degree granting college to higher university degree (MA)	3–80 years (majority > 16 years)	0–4	3 of 12
Young adults	21–28 years	Six single, one engaged, one cohabitant, and two in a relationship	Upper secondary school to lower university level	6 months to 11 years (majority < 3 years)	0	3 of 10
**India**						
Older adults	60–82 years	Six widow(er) and six married	Basic school to Ph.D.	1–50 + years (majority > 10 years)	0–3	8 of 12
Young adults	18–25 years	10 of 10 single	College to lower university level	1–21 years (majority < 2 years)	0	5 of 10

### Interview

In PSOC studies, the interview method has been used to gather information in diverse cultural contexts (e.g., Afghanistan (Brodsky, 2009) and Australia (Bishop et al., 2006)). A semi-structured interview form was chosen, to ensure an overall structure to the interview while at the same time giving informants a possibility to express their personal understandings of PSOC. The interview guide consisted of two sections; Part 1 covered the background information (age, gender, marital status, level of education, residential area of Mumbai/Oslo, years of residence, number of children, number of family members residing in their home, and social and political participation/membership). Part 2 included questions about the meanings of community and PSOC, such as: “What comes to your mind when you think of the word ‘community’?”, “What meaning do you relate to the concept ‘sense of community’?”, “What characteristics do you think a ‘good community’ should have?”, and “Do you have any experience of past members of the community being excluded or leaving the community?”

All interviews were conducted by the first author in Norwegian in Norway, and in English in India. Although English is the central part of several Indian languages, a translator assisted the Norwegian-Indian first author in the interviews in India, to ensure that the questions were understood as intended. The interview settings (the senior centres/day care centres, the campuses, the homes of the informants, or the apartments of the interviewers) were chosen by the informants. All interviews were recorded (mean length = 49 min for the older adult samples, and 42 min for the young adult samples) and transcribed.

### Ethical Considerations

All the interviewed informants were informed about the purpose of the study and gave their written consent before the interviews were conducted. Also, before interviews were conducted, the research was approved by the Norwegian Ethics Committee (The Norwegian Centre for Research Data) owned by the Norwegian Ministry of Education and Research. No further ethical approval was required for this study as per institutional and national guidelines and regulations.

### Analyses

In this study, we used summative content analysis. Typically, a first step is conducted to identify and quantify codes (words and expressions) and then a second step is done by describing and interpreting codes with respect to their context of use (Hsieh and Shannon, 2005; Vaismoradi et al., 2013). We first conducted preparations of the material so that we secondly could perform quantitative analyses to identify words people use when describing PSOC, words which are also particularly interesting for qualitative in depth analyses of PSOC. Then, as a third step, we did qualitative in-depth analyses of the use of these particularly interesting PSOC words with respect to their context of use (local and global meaning systems).

### Preparations for the Analyses

As the first step in our analysis, we read through the interview material (Part 2) and identified words and expressions our informants used to define and describe PSOC. We took care to sample words used within each of the four sub-samples (young adults and older adults from each cultural context), in addition to words used across all the four sub-samples. We then made text search queries for the identified words in NVivo. Additional NVivo text search queries were made for words included in operationalisations of PSOC, e.g., “accept,” “share,” and “need,” including negative PSOC, e.g., “problem,” “frustrating,” and “different” (McMillan and Chavis, 1986; Brodsky et al., 2002; Mannarini et al., 2014). Every NVivo text search provided information on how many times each word was used by each informant and sub-group, and where in the interview text the word was located.

Altogether, we undertook NVivo text searches for 69 words or expressions. Next, we deleted search words which were predominately used to describe aspects unrelated to PSOC (e.g., the word “get” as used in, “I get the impression that…”); words which were meaningfully ambiguous (e.g., “right,” which holds several meanings); or words which had very few (less than 2) cases of usage for all the four sub-samples. We were then left with 19 positive PSOC words and 17 negative PSOC words. For these included words, we removed cases of usage not relevant to community or PSOC descriptions (e.g., the word “different” as used in, “Now you are asking a different question”). To ensure satisfactory breadth in the analysis, inclusion criteria for the search words to be further analysed were that the words and expressions should be used by half or more of the informants. Out of the 19 positive PSOC words and expressions, seven words (“family,” “help,” “care,” “each other,” “give,” “interest,” and “respect”) met this criterion. Of the 17 negative PSOC words, only two met this criterion (“different” and “problem”) (see Table 2).

**TABLE 2:** Frequency table for selected words sorted by country and age groups.

	**Norway (n22)**	**Adolescent (n10)**	**Older adult (n12)**	**India (n22)**	**Adolescent (n10)**	**Older adult (n12)**
Family	90 (18)	45 (9)	45 (9)	77 (16)	44 (7)	33 (9)
Help	46 (16)	4 (4)	42 (12)	126 (18)	57 (8)	69 (10)
Care	9 (6)	1 (1)	8 (5)	41 (13)	7 (4)	34 (9)
Each other	47 (21)	33 (9)	44 (12)	59 (16)	22 (9)	37 (7)
Give	56 (16)	11 (7)	45 (9)	86 (16)	36 (8)	50 (8)
Interest	47 (13)	14 (7)	33 (6)	20 (7)	6 (2)	14 (5)
Respect	25 (12)	15 (6)	10 (6)	51 (16)	22 (7)	29 (9)
Problem	29 (6)	3 (2)	17 (4)	46 (15)	20 (9)	26 (8)
Different	44 (12)	11 (6)	33 (6)	163 (21)	101 (10)	62 (11)

*Number of informants in brackets.*

### Quantitative Analysis

Chi-square tests were used to explore the relationship between the use of a word (number of informants using a specific word) and age and cultural context. To avoid compromising the low statistical power due to low *n*, only bivariate associations in 2 × 2 tables were explored, i.e., the analysis did only discriminate between used and unused words, and did not take into account, the number of times the word has been used. This was undertaken to ensure valid chi-square tests (i.e., sufficient expected counts in cells). To assess the strength of associations and to enable easy comparison, the effect size Φ was used. The value, Φ = 0.10 represents a small effect size, 0.30 represents a medium, and 0.50 a large effect size (Cohen, 1992). The IBM SPSS version 23 was used to perform these analyses.

As presented, the aim of the quantitative analysis was to identify words and expressions that were relevant for further qualitative analyses to a more systematically and in-depth analysis to uncover the PSOC concept in the two different life-stages; both of them at a risk for rather strong, ordinary life stress, and from two different cultures. Non-parametric analyses can be used to analyse broad classifications (such as the definitions of PSOC by informants) from interview data, where all informants have been asked the same questions (Morse, 2005).

No significant relationships were observed between cultural context and the use of the words of central relationships (“family,” “help,” “care,” “each other,” “give,” “interest,” and “respect”). But there was a significant overrepresentation of informants from India using the word “care,” [χ^2^ (1) = 4.54, *p* < 0.04, Φ = 0.32] and a significant overrepresentation of informants from Norway using the expression “each other,” [χ^2^ (1) = 4.25, *p* < 0.05, Φ = −0.31]. Compared to Norway, more informants from India used the word, “different,” [χ^2^ (1) = 7.33, *p* < 0.01, Φ = 0.41], and “problem,” [χ^2^ (1) = 7.38, *p* < 0.01, Φ = 0.41) when asked about PSOC.

When comparing the two age groups, both the young and old adult, no significant relationships were observed regarding the words, “family,” “each other,” “give,” “interest,” “respect,” “problem,” and “different.” However, the word, “help” was used more often by older adult informants in both cultures than by the young adults [χ^2^ (1) = 6.23, *p* < 0.02, Φ = 0.38]. The same was the case for “care,” [χ^2^ (1) = 4.94, *p* < 0.03, Φ = 0.34].

### Qualitative Analysis

Based on the above statistical analyses, five of the words were chosen for an in-depth qualitative analysis: the two positive PSOC words, “care” and “help” were central as well as the two negative PSOC words, “problem” and “different.” Finally, the most clearly community-related word, “family,” used globally, was included. This word demonstrated strong similarity in usage: high frequencies of usage across all the four sub-samples. Moreover, the usage was equally distributed across age and culture.

By means of text search queries in NVivo we retrieved all segments of the interviews where any one of these five words occurred to conduct a second analysis; a qualitative analysis of these particular text segments. This second analysis included (1) reading the excerpts for each of the four sub-groups, (2) writing a brief report summarising how each of the sub-groups used and/or spoke about each of the five selected words, (3) identifying the differences and similarities in the ways the different sub-samples used these words, and finally (4) presenting and interpreting the findings with respect to life-stages and cultural meaning systems.

Informants are represented by codes indicating gender (F, female; M, male), cultural context (N, Norway; I, India), and age. Four informants in the Norwegian sample were 85 years old, and they were given an additional number (1–4) to distinguish them.

## Qualitative Findings: Analyses Based on the Selected Words Identified Through the Quantitative Mapping

### “Help” and “Care”

“Help” and “care” were central for all groups in the concept of PSOC. The statistical analysis, however, demonstrated that the two words, “help” and “care” were used more by older adults compared to younger adults when describing PSOC. Moreover, Norwegian older adults differed from the other three sub-samples in referring also to the macro-level of the society; public health system— doctors, hospitals, social workers, and psychologists—when talking about “help” and “care.” For them, PSOC was also a meaning system about “help” and “care” from society at large.

MN75: “…they have social workers here in my community [the senior centre]. Which…eh…I have got help from.”

Indian older adults, on the other hand, described “helping” primarily as their own responsibility or obligation to invest in community development also at the city and macro levels:

FI82: “…community development has to take place… And whosoever who can help, they should pull out their resources, their energy, their time, and money…and make that community [Mumbai] worthwhile living.”

The older adults in Norway mentioned this type of social responsibility too, but their descriptions were predominately about securing their own and the PSOC of their peers, not investing in the community at large. This finding was underpinned by the analysis of the word, “problem”; for the older Norwegians, this word was not used about their investment in the larger community, as it was for the Indian older adults.

Both in Oslo and Mumbai, the young adult informants defined their near social context —student groups and the campus— as the central communities both for receiving as well as giving help or support:

MI25: “Yes, I have [a sense of community] in Mumbai. Especially in Tata Institute of Social Sciences …Teachers are very helpful. Staffs, all the staffs are very helpful…”

MN21: “I’m in this student group which I participate quite a lot in…I just participate and help out a little…”

All four sub-samples then used the words, “Help” and “Care” when asked about their meaning of community and PSOC.

FN851: “Yes. Care [is an important element of community]. As in kindness.”

FI21: “Sense of community to me would mean sense of belonging…and a place or group of people where you can really speak up your mind, and expect some kind of approval and acceptance and help from the community.”

“Help” and “care” were the words used by all the four sub-groups as parts of their meanings of PSOC. Despite being more often addressed by the two older adult samples, the qualitative analysis highlighted similarities across the four sub-samples of the value of help and care. At the same time, the findings demonstrate core aspects of each of the cultural contexts as reflected in how young and older adults define and describe help and care as part of PSOC: the two Norwegian samples describe PSOC in terms of securing own personal needs, primarily at the individual level, not mentioning the individual responsibility at the macro-level. The two samples from India, on the other hand, describe PSOC as care in ways reflecting collectivistic values: helping others and the larger community. The older adults in Norway, on the other hand, was the only group describing help in terms of society’s care for protecting and helping citizens through their life course. This reflects the Norwegian societal context: a strong public welfare ideology. People are all citizens of society at large and the state is conceived as a central provider for the health and economic situation of the people, particularly in old age (Daatland and Herlofson, 2004). Finally, the finding of the near social context as the central community for the young adults in India and Norway receiving and giving help demonstrates the core aspects of the situation of the young adults: transitions, moving from the family to a new community. This is the situation for young adults across cultures (Arnett, 2002; Mahan et al., 2002).

### “Different”

The quantitative analysis of the use of words by the informants showed that the negative PSOC word, “different” was used more frequently describing PSOC by the Indian than the Norwegian samples. Through the qualitative analysis, we identified that the word was used by the two Indian sub-samples in a similar way:

MI60: “Community means that it is a group of people belonging to different race, different caste, different regions, but all are living happy.”

FI23: “…a good community will be a good mix of different people…in terms of gender, class, religion, and…. I think…that enriches the experience of a community.”

The qualitative findings confirmed the word, “different” as the central part of the meanings of PSOC of both young and older Indian adults: PSOC is also about the inclusion of “difference.” A good mix of people is a central part of a society’s wellbeing. No such trend was found in the Norwegian usage of the word.

With respect to the findings for the urban Indian samples, we interpret these findings as reflecting the heterogenic meaning systems of PSOC within their cosmopolitan city context (Bahl and Hagen, 2017). Key characteristic of the community for the two Indian groups is also about diversity. The Norwegian samples did not use the word, “different” in their descriptions of PSOC indicating that heterogeneity may not be an important aspect of their conceptualisations of PSOC.

### “Problem”

The negative PSOC word, “problem” was statistically identified as a word used more frequently by the two Indian samples. Moreover, Indian older adults were different from the other three sub-samples by addressing one particular type of problem, “family problems,” with detrimental consequences for where to belong, particularly losing the possibility of living with their family:

MI81: “… as I told you, most of them come [to old age homes] because of family problems. Earlier, India was famous for group family living, it is no more…”

Changes in the society and family structures as well as the new practice of placing senior citizens in old age homes were frequently addressed; destroying family belongingness and thereby destroying the wellbeing in the last period of life.

The young adults from India were the only sub-sample who expressed inter-generational problems of membership when conceptualising PSOC:

FI23: “I always had my views that…what the elderly are doing to our community is wrong, “what do they know about what the young want…”…and now, because of all these problems within the community I feel that a lot of the young are moving away from it [the community]…”

Obviously, the two Indian age sub-groups being in different phases of life experience very differential lives and communities, which is important for their PSOC.

Young and older Norwegian adults were similar in the way they talked about problems. They talked particularly about individual needs.

MN21: “It’s more like you have to…you have to be independent and do everything yourself, and then there is of course more problems to be handled. I have definitely had more use of the community now than before [to fulfil my needs].”

FN851: “A person like me can also be strained. Because I have problems with restricting…It becomes too much… So I have to, have to put up boundaries around me…You have to take care of yourself too.”

At the same time, some older adults seem to miss being part of supporting and caring social networks.

Like the older adult Indian sample, young adult Indian informants were also concerned about the community problems; one has to take responsibility to care for different communities, for example, their city, as had happened some years ago.

FI82: “…another characteristic of a community is that whosoever needs help, should be given help by…and his problem should be sorted out by the community around him.”

MI18: “…if there is some problem in a group of people then the community helps out, that way. As in, there where floods in Mumbai a couple of years ago, and at that time many people had actually helped people by carrying them out in the rain, they were giving them food, etcetera, they were taking care of them. So at that time, it was like Mumbai was active like a community.”

As shown, there were marked similarities and differences in how the word, “problem” was used by the two Indian age groups, as well as across the two cultural contexts. The findings illustrate clearly the Norwegian meanings of PSOC as individualistic and Indian meanings as collectivistic. At the same time, the family problems and inter-generational issues of concern for the Indian samples also point to a more individualistic meaning system growing in strength; particularly, the nuclear family structure rests currently more and more on the values of strong individualism (Bhat and Dhruvarajan, 2001; Ansari, 2007). The increasingly globalising individualistic values, conflicting with traditional collectivistic values, as in urban India, result in different meaning systems among the young and old with consequences for the feeling of people as part of the community, of belonging (Shah, 2009). To sum up, the vision of the Indian young persons with regard to family structure and citizenship is no longer automatic in solidarity with the traditional practice of living together with and taking care of the older. In neo-liberalist urban societies, family structures have been fragmented, resulting in families living apart, as in individualistic Norway.

### “Family”

Among social relationships, family relationships are ranked as the most stable source for the basic need to belong (Baumeister and Leary, 1995; Lambert et al., 2010). In a cross-cultural study, mapping lay definitions of life satisfaction in 12 different nations around the world (including both India and Norway), interpersonal relationships and family were also the most frequently mentioned contextual factors in lay definitions of life satisfaction (Delle Fave et al., 2016).

“Family” (and “help”) was also the most frequently used word chosen for qualitative analysis. All four sub-samples used the word, “family” in describing PSOC.

Both young and older Norwegians talked about family as one of the most important communities overall:

MN80: “My strongest belonging is of course within the family.”

MN26: “Most important [community for my PSOC] …well. I think it has been and will always be the family.”

The Indian sub-samples were in addition concerned with the position of the family (vertical) in the society.

MI21: “Apart from that, my caste, my religion, and my family… that, actually right from your birth you get a status, ok? And…that status gives you too much of an opportunity also, ok? And that actually is the first relation between you and the community?”

FI67: “I [experience my community as a community] just… because, this is the area where I could see certain…something common, like the people have something common here, and this is maybe age-wise, family status, you know…”

As shown, by the two Indian samples, the family was regarded central in affecting and defining how the individual family members were perceived and treated by the larger society.

Families are of different types. Although “family” was a word used by all four sub-samples when describing PSOC, the qualitative analysis revealed different ways of using the word within the two social contexts illustrating the core cultural aspects of the Indian and Norwegian cultures, respectively; the Norwegian samples described the family as important for own PSOC, while the Indian samples were concerned more with PSOC as interrelated with the PSOC of the family in the larger community, thus reflecting traditional differences between collectivistic and individualistic societies.

The importance of the family unit in collectivistic societies has been strong (Chiu and Hong, 2013) and the family has been understood as the central community and source for the PSOC of people in collectivistic cultures (Brodsky, 2009; Cicognani et al., 2014; Mannarini and Rochira, 2014; Carrillo et al., 2015). Older adults in India also mentioned “family” markedly more frequently than older adults in Norway. This cultural difference, however, disappeared when young adults from India and Norway were compared. Such a change across generations may reflect the current globalisation of the ongoing neo-liberalist ideology.

## Summary and Discussion

The present study showed that when describing the meanings of PSOC, young and older adult informants from the individualistic Norwegian culture tended to use words—related to positive and negative aspects of PSOC (“help” and “problem”)—with reference to the individual level, while informants from the same age groups in the collectivistic Indian culture had a tendency to use the very same words in terms of the larger community. These findings are in line with the earlier findings suggesting a relationship between the two cultural syndromes and meanings of PSOC (Sarason, 1974; Love, 2007; Brodsky, 2009; Nafstad et al., 2009; Moscardino et al., 2010; Bahl and Hagen, 2017; Bahl et al., 2017). Moreover, how the family was spoken about also clearly reflected the cultural syndromes of the two contexts, consistent with other findings for the PSOC of young adults (Moscardino et al., 2010; Cicognani et al., 2014). These findings indicate that the associations between PSOC, family, and cultural syndromes are central to understand the phases of life both in India and Norway.

The study also showed some important life-stage related aspects of PSOC. In the Norwegian context, these aspects were specific to the cultural context; “help” and “care” as part of later-life PSOC reflected the Norwegian welfare state ideology. In the Indian context, on the other hand, the age-specific aspects—the problems raised by older adults about the family structure and by young adults about intergenerational community relations—showed how both local and global meaning systems influence within the Indian urban context; the co-existence of collectivistic as well as individualistic meaning systems. The fact that Norwegian samples define the family as a community, which has previously been reported in PSOC studies from collectivistic cultures, and that individualism is revealing in communities as well as in the social structures of urban India—particularly in the heterogenic and cosmopolitan context of Mumbai—suggests that current globalisation makes the meaning systems in the societies more alike.

As pointed out, different life-stages entail specific psychosocial transitions, which affect PSOC (Phillipson, 1993; Fyson, 2008; Chiessi et al., 2010; Cicognani et al., 2014; Bahl et al., 2017). The findings suggest that some transitions, e.g., those for young adult people, are more shaped by the dominating social roles and neo-liberal meaning systems of cultures, while transitions in old age are more changing, variable, and locally context-dependent.

To sum up, young adults as well as older adults in urban Norway and India negotiate challenging psychosocial transitions affecting their PSOC. However, the reorientation of older adults to psychosocial transitions—retiring and withdrawing from communities and loss of social relationships, as well as changes in meaning systems, from the ones they grew up with to the emerging and sometimes conflicting contemporary ones—demand greater efforts and activity from the individual to adapt, stay socially involved, and maintain their PSOC in the later-life. Then, the policy and practice that aim to promote health and wellbeing across the life-span need to acknowledge that central meaning systems are embedded in cultural contexts forming and shaping the PSOC of the people. Finally, the findings indicate that stronger individual effort is needed to ensure own PSOC in old age compared to that in the younger phases of life both in Norway and India. Both transitions in old age and changing meaning systems represent challenges for the PSOC of older adults. As such, it is especially important that policy and health professionals in these contexts facilitate and empower the individual effort of the older adults in securing their own PSOC in everyday life.

### Challenges for Community Psychology and Applied Social Sciences: The Need for Ongoing “We” Discourses in This Special Historical Time

Is there a core meaning system of PSOC shared by people within as well as across cultures? As shown, the meaning system of PSOC for all four groups was about people, primarily of the family. The word, “family” demonstrated high frequencies of use across all sub-samples. There was nothing of more relevance for the four groups than the family system when thinking of PSOC; family was about belonging, problems, lack of, and missing. The PSOC meaning systems of the four groups reveals almost an outside of society or, more precisely, a “prior-to-society-perspective,” when thinking of PSOC (Damon, 1983). PSOC in Mumbai and Oslo is in one way or another about being part of a family. People from India or Norway, young or older adults, during good or difficult phases of life, are members of families. Family remains as the core of PSOC shared by people of different ages as well as across cultures. The concept of PSOC, then, is more about the private, not the public sphere. At the same time, however, we are members of other groups and communities; schools, workplaces, neighbourhoods, organisations, cities, and nations. The four groups mentioned such communities and groups, but less often.

Feeling part of, helping and caring for your neighbourhood, your village, your town, or national and global communities, not only the family, are necessary; particularly today, when we find ourselves in a historical time where communities, societies, and nations all over the world are being heavily impacted by the COVID-19 pandemic^1^. The most crucial task for community psychology today is to highlight and maintain the current feeling of connections by people, belonging, and social responsibility not only for the family but also for other communities and for other societies. Community psychology then has to systematically articulate and promote discourses of bonding and social responsibility for multiple communities: neighbourhood, working place, and communities and society at large. PSOC has traditionally been a predictor for local citizen participation, particularly in geographical communities, such as neighbourhoods (Perkins and Long, 2002). It is, however, now imperative with broader and multifaceted discourses of the commitment of the society to the community. Community psychology and applied social sciences have to mobilise and foster community engagement and social responsibility at all levels in society—also globally. A necessary task for community psychology and the applied social sciences, then, is to assist and join stakeholders, communities, municipalities, governments, and global health organisations in co-creating intervention programmes to mobilise, foster, and promote citizen engagement and the solidarity of national and transnational citizenship.

The findings of the present study point to the usefulness for community psychology and the social sciences of providing and using a variety of concepts of social connections. However, as presented, all over the world, there has been a growing individualism, what we can call a more excessive individualism being prioritised (Halpern, 2005; Nafstad et al., 2009, 2013; McDonald et al., 2017; Prilleltensky, 2020). The influence of an increasingly powerful globalising neo-liberalism changes, as also shown in the present study, societies in individualistic as well as collectivistic cultures toward more individualism, with profound implications for social structures, social life, and the feelings of togetherness by individuals and their ability in coping across the life span (Bauman, 2001; Fairclough, 2006; Nafstad et al., 2009). We are, as Twenge and Campbell (2009) pointed out, living in a historical time of strong “me” culture. Given the predominant ideology of our era, more and more has been the idea of the lonely citizen managing all/everything by her/himself or her/his nearest family; this enterprise of constructing togetherness then is a very labour-intensive process indeed. However, this enterprise is highly necessary, for example, today at this time of the ongoing corona epidemic. As Jovchelovitch (2007: 71) concludes, “Togetherness is a long and labour intensive process that needs to be constructed; it is an achievement”. Public policy programmes of how to take responsibility and care for others, not only for oneself and the family, have continuously to be co-created with stakeholders and empirically evaluated by community psychology, with respect to the traditional and socially just aim of wellbeing for all (Sarason, 1986; Von Heimburg and Ness, 2020).

As presented, words reflect thoughts, feelings, and values (Nafstad et al., 2009; Moghaddam and Harré, 2010; Normann, 2019). Often overlooked, the words and concepts we use are the medium through which we develop our sense of community, social responsibility, and our willingness to be part of. Today the word, “we,” the sense of “we,” more than before has to be used and expanded to ensure life, health, and wellbeing. For example, individual health and wellbeing are deeply dependent on our sense of solidarity with all citizens, not only to particular groups in a society or a nation; we are all in this global pandemic. Words and phrases, such as “all nations,” “families of nations,” “universal care and help systems,” “global solidarity,” “civic duty,” “civic responsibility,” “citizen engagement,” “we are all in this together,” etc., have now to be integrated into parts of our ongoing discourses. To conclude, currently a most important challenge for community psychology is to develop a variety of concepts of belonging, demonstrating both our collective and our individualised connections.

## Limitations, Strengths, and Future Studies

Life course research centres on generational similarities and differences, on psychosocial transitions and age-related meaning systems in cultural-historical contexts. In this study, we aimed to provide a multifaceted understanding of PSOC and culture in India and Norway; how interactive meaning systems of local and global values within these two cultural contexts are reflected in the meaning systems of PSOC among two age groups which are in different life-stages. There are several limitations and strengths within the study, which should be addressed and highlighted with respect to future research.

First, to ensure the ecological validity of the study, this study included theory on the meaning systems surrounding the samples to understand their discourses of PSOC as embedded in each of the respective cultures. To increase the sensitivity of the study to the context it may be fruitful to include macro-level data (on public discourses) and not only to micro-level data. Secondly, to ensure a satisfactory breadth and depth to the analysis of meaning systems of PSOC, we included four samples of informants with different demographic backgrounds and in different situations (see Table 1). This was essential to ensure a level of multivocality and credibility of the findings (Tracy and Hinrichs, 2017). Our settings of recruitment could have easily restricted the breadth of the samples: Recruiting the young adult informants at university campuses and through acquaintances could have resulted in restricted use of words by socio-economic groups in describing the PSOC. Recruiting old adult informants from senior centres could, in the same manner, have resulted in a particular group of old people; rather than independent and healthy people. However, using a combination of purposive (recruiting older adults from different parts of the two city contexts) and convenient (recruiting young adults who randomly passed the first author at the two campuses in addition to snowball sampling) recruiting strategies, most likely secured samples with different demographical backgrounds and in different situations. Future research should test out additional combinations of purposive and convenient sampling strategies to ensure even broader samples of both young and old adults, as the meaning systems of people can be more or less connected to specific local socio-political values and ideals.

Finally, the use of summative content analysis provided the possibility to explore the material in a broader and systematic way as well as to go deep into the material with respect to differences and similarities in the meaning systems of PSOC. However, our analysis of meaning systems as embedded in local values is largely dependent on the bi-cultural background of the first author, as Norwegian-Indian. We assume that future research on the PSOC meaning systems of people as embedded in interactive meaning systems of local and global values will benefit from utilising this kind of bi-cultural asset in future research on the meaning systems of PSOC.

## Conclusion

Our findings suggest that several meaning systems of PSOC can co-exist within as well as across cultures; the findings showed that when describing meanings of PSOC, young and older adult informants from the individualistic Norwegian culture tended to use words —related to positive and negative aspects of PSOC (“help” and “problem”)—with reference to the individual level, while informants from the same age groups in the collectivistic Indian culture had a tendency to use the very same words in terms of the larger community.

Moreover, the findings indicate that meaning systems of PSOC and citizenship vary according to different age groups and cultures; the study showed some important life-stage related aspects of PSOC. In the Norwegian context, these aspects were specific to the cultural context; “Help” and “care” as part of later-life PSOC reflected the Norwegian welfare state ideology. In the Indian context, on the other hand, the age-specific aspects— the problems raised by older adults about the family structure and by young adults about intergenerational community relations— showed how both local and global meaning systems influence within the Indian urban context; the co-existence of collectivistic as well as individualistic meaning systems.

At the same time, the findings suggest that the meaning systems of people are greatly influenced by the ideological climate of neo-liberal globalisation and as a result, becoming more alike; the fact that Norwegian samples define the family as a community, which has been reported only previously in PSOC studies from collectivistic cultures, and that individualism reveals in communities as well as in the social structures of urban India – particularly in the heterogenic and cosmopolitan context of Mumbai – suggests that current globalisation makes the meaning systems of the societies more alike.

To conclude, sense of togetherness and citizen participation are ultimately an ongoing and negotiating process and togetherness is a process of hard work across the life span. Today, as pointed out, we find ourselves in a special historical time—a virus pandemic all over the world—that has activated meanings systems also of a more global “we.” Currently, as addressed, this situation provides a unique possibility for community psychology and applied social sciences to co-create and set alive transformative multi-level research and interventions (e.g., public policy programmes) promoting and maintaining the feeling of connections and social responsibility of people, broadly at all levels in the society—also globally. Most importantly, with meaning systems as the point of departure, research and interventions will be context-sensitive having an increased likelihood of being transformative—also in the future, when the pandemic ends and the neo-liberal ideology goes on.

## Data Availability Statement

The raw data supporting the conclusions of this article will be made available by the authors, without undue reservation.

## Ethics Statement

The studies involving human participants were reviewed and approved by the Norwegian Centre for Research Data. The patients/participants provided their written informed consent to participate in this study.

## Author Contributions

NKHB: has the main responsibility of planning the overall study, conducting the analyses, interpretation of data, and writing and leading the collaboration behind the manuscript. HEN and RMB: has collaborated in writing the manuscript, giving advice on analyses and contributed with suggestions for literature, and also contributed greatly in writing about the challenges for community psychology and applied social sciences. EL: has collaborated in the statistical analysis of the study and participated in writing the manuscript. All authors contributed to the article and approved the submitted version.

## Conflict of Interest

The authors declare that the research was conducted in the absence of any commercial or financial relationships that could be construed as a potential conflict of interest.

## Publisher’s Note

All claims expressed in this article are solely those of the authors and do not necessarily represent those of their affiliated organizations, or those of the publisher, the editors and the reviewers. Any product that may be evaluated in this article, or claim that may be made by its manufacturer, is not guaranteed or endorsed by the publisher.

## Footnotes

1 The data for this study were collected prior to the COVID-19 pandemic. However, given the clear relevance of our findings to this special historical time, we have chosen to utilise the findings with respect to this global context and the special issue that the article is part of by suggesting some central challenges for community psychology and the applied social sciences.
